# HIF1*α* is dispensable for oocyte development and female fertility in mice

**DOI:** 10.7717/peerj.13370

**Published:** 2022-05-03

**Authors:** Yujia Chen, Siyu Du, Zhenyue Huang, Longsen Han, Qiang Wang

**Affiliations:** 1 State Key Laboratory of Reproductive Medicine, Suzhou Municipal Hospital, Nanjing Medical University, Nanjing, China; 2 Center for Global Health, School of Public Health, Nanjing Medical University, Nanjing, China

**Keywords:** HIF, Hypoxia, Oocyte, Embryo, Fertility

## Abstract

**Background:**

It has been thought that oocyte may develop in a low oxygen environment, as changes in follicle structure and formation of a fluid-filled antrum. The survival of hypoxic tissues is controlled by hypoxia-inducible factors (HIFs) that are activated in a low oxygen state. HIF1*α* is expressed in mature mouse oocytes and continues to be expressed after fertilization, from the 2-cell to blastocyst stage. However, the physiological roles of HIF pathway during oogenesis and embryogenesis have still not been elucidated in detail.

**Methods:**

Mutant mice with oocyte-specific HIF1*α* deletion were generated by crossing *Hif*1*α*^*fl*/*fl*^ mice with transgenic mice expressing *Gdf9*-promoter-mediated Cre recombinase. Breeding assay was carried out to detect female fertility. *In vitro* fertilization and embryo culture were used to assess early embryo development. Oocyte meiotic progression was also examined. Quantitative RT-PCR was used for analyzing of candidate genes expression.

**Results:**

We successfully generated mutant mice with oocyte-specific deletion of HIF1*α*. Oocytes loss of HIF1*α* did not affect female fertility, ovulation and early embryo development. Moreover, oocytes can mature in vitro, and form well-organized spindle in the absence of HIF1*α*. In addition, pronounced differences in *Hif2α* and *Hif3α* mRNA expression were not observed in HIF1*α*-deleted oocytes. These results revealed that HIF pathway in oocytes is not essential for female fertility.

## Introduction

Oxygen occupies a central role in the maintenance of life as we known it, perhaps most prominently in aerobic metabolism, where O_2_ serves as the terminal electron acceptor in oxidative phosphorylation (Kaelin Jr & Ratcliffe, 2008). Inadequate oxygen availability can lead to cellular dysfunction and, if sufficiently profound, cell death, in aerobic organisms (Kaelin Jr & Ratcliffe, 2008). As organisms become larger and more active, oxygen transport by simple diffusion becomes limiting. To maintain oxygen homeostasis, mammals have evolved specialized networks to maintain oxygen homeostasis at the tissue level. One of the critical aspects of this network is oxygen-dependent posttranslational hydroxylation of a transcription factor called hypoxia-inducible factor (HIF) (Giaccia, Simon & Johnson, 2004; Kaelin Jr & Ratcliffe, 2008). HIF is a heterodimer of bHLH-PAS (basic-helix-loop-helix, per-ARNT sim) proteins and consists of an unstable *α*-subunit and a constitutively and ubiquitously expressed *β*-subunit HIF1*β* (Bruick, 2003; Semenza, 2001). HIF*α* subunits exist as a series of isoforms encoded by distinct genetic loci. Three HIF*α* proteins have been found in higher metazoans, and HIF1*α* and HIF2*α* are able to interact with hypoxia response elements to activate transcription. Under well-oxygenated conditions, hydroxylation of one (or both) of two highly conserved prolyl residues located near the NTAD by prolyl hydroxylase domain-containing (PHD) proteins mediates interactions with the von Hippel-Lindau (VHL) E3 ubiquitin ligase complex that targets HIF*α* for proteasomal destruction (Ivan et al., 2001; Maxwell et al., 1999). Accordingly, hypoxia can inhibit HIF*α* hydroxylation, which leads to HIF*α* accumulation and nuclear translocation, and consequently activating genes involving in widespread biological processes (Kaelin Jr & Ratcliffe, 2008).

The ovarian follicle provides the oocyte with ideal environment for growth and development in preparation for ovulation and fertilization. In ovary, as the follicle enlarges, a basement membrane separates theca and granulosa cell layers, and thus antral follicles are not directly adjacent to vascular support. In addition, the formation of a fluid-filled antrum physically separates the cumulus-oocyte complex (COC) from granulosa layers (Redding, Bronlund & Hart, 2007; Rodgers & Irving-Rodgers, 2010). Consequently, the oocyte itself might develop in a potential hypoxia environment. There exists a paradox in that while the maturing oocyte resides in an avascular environment, it also relies heavily on oxidative phosphorylation which requires oxygen (Thompson et al., 2015). The activation of HIF target genes had been implicated in resolving this paradox. During follicle development and corpus formation, VEGF (vascular endothelial growth factor), the main inducer of angiogenesis, was upregulated by HIF. In addition, ovulation is also intrinsically linked to HIF activity through the ovulatory luteinizing hormone surge increasing HIF expression. Furthermore, HIF1*α* is presented in mature mouse oocytes and continues to be expressed from fertilization to blastocyst stage (Takahashi et al., 2016).

However, the physiological roles of HIF pathway during oogenesis and embryogenesis have still not been elucidated in detail. In this study, we delete *Hif*1*α* specifically in oocytes by *Cre-loxp* conditional knockout (Yu et al., 2013) and show that HIF1*α* deletion exerts little effect on oocyte maturation and embryo development *in vivo* and *in vitro*.

## Materials and Methods

### Mice

Animal care and use were carried out in accordance with the guiding principles of the Institutional Animal Care and Use Committee (IACUC) of Nanjing Medical University (Approval No. IACUC-1806013). Mice housed in 12–12 h light-dark cycle, with constant temperature and with food and water provided *ad libitum* under SPF (specified pathogen free) conditions. Mice were randomly divided into cages, and each cage was capable of housing 4–5 mice. All cages were maintained in similar conditions, including cages density, bedding, and sanitation frequency. Mice were anesthetized with carbon dioxide for oocyte collection. No animals survived at the end of study.

Mice possessing *loxP* sites flanking exon 2 of the *Hif1α* gene were kindly provided by Dr. Jin Hou at Second Military Medical University (Liu et al., 2019). *Gdf9-Cre* transgenic mice were a gift from Dr. Heng-Yu Fan (Yu et al., 2013). To generate *Hif1α*^*fl*/*fl*^; *Gdf9-Cre* mice (referred to as *Hif1α*-*cKO*), female mice carrying the *Hif1α* floxed alleles were mated with *Gdf9-Cre* males (Yu et al., 2013). The *Hif1 α*^*fl*/*fl*^ female mice were used as the control group (referred to as Control). Genotyping for LoxP and Cre were carried out using PCR amplification. Primers for *Hif1α Loxp* (Forward: 5′—AGTTACAGGTATTTATGAGCCA—3′, Reverse: 5′—CTAGTTGATCTTTCCGAGGAC—3′), and *Gdf9-Cre* (Forward: 5′—GGCATGCTTGAGGTCTGATTAC—3′, Reverse: 5′—CAGGTTTTGGTGCACAGTCA—3′) were used at 10 pmol using Vazyme PCR mix following manufacture’s protocol.

### Fertility test

To perform fertility test, seven pairs of 8 weeks Control and *Hif1α*-cKO female mice were randomly selected and continually bred with WT male mice which have been confirmed fertility for 6 months. The mice were checked every week and the date and number of pups and litter size was recorded for each litter.

### Oocyte collection and *in vitro* maturation

To collect GV oocytes, 6-8 weeks of females (*n* = 5) were stimulated with 5 IU PMSG (pregnant mare serum gonadotropin) (Ningbo, No. 2 hormone factory, Zhejiang, China). After 44–48 h, GV oocytes were carefully isolated from antral ovarian follicles by manual rupturing of antral ovarian follicles. For *in vitro* maturation, Oocytes were cultured in M2 media under mineral oil at 37 °C in a 5% of CO_2_ incubator.

To obtain MII oocytes, mice (*n* = 5) were induced to superovulate by IP injection of 5 IU PMSG followed 48 h later by injection of 5 IU hCG (Human Chorionic Gonadotropin). 16 h after hCG injection, mice were sacrificed and the oviducal ampullae were broken to release the cumulus-oocyte complexes. MII oocytes were freed of cumulus cells by exposure to 0.2% hyaluronidase.

### *In vitro* fertilization and embryo culture

IVF assays were conducted as we described previously (Han et al., 2018). Briefly, normal sperm were isolated from the dissected epididymis of C57BL/6 male mice aged 10–20 weeks and left to capacitate for 1 h in HTF fertilization medium (Millipore, Merck) supplemented with 10 mg/ml BSA (bull serum albumin). Cumulus–oocyte complexes (COCs) were isolated from oviduct ampullae, and placed in other HTF fertilization medium (Millipore, Merck) supplemented with 10 mg/ml BSA. Then, dispersed spermatozoa were added to HTF drops containing COCs for fertilization in a 37 °C incubator. After co-incubation for 6∼9 h, presumptive zygotes were washed to remove cumulus cells and excess sperm, and then transferred into KSOM medium (Merck Millipore, Burlington, MA, USA) for further culture. Early embryo development potential was assessed at the indicated time points during culture.

### Western blot

Samples each containing 100 MII oocytes from 5 mice were collected in SDS sample buffer and heated for 5 min at 100 °C. The proteins were separated by 10% SDS-PAGE and then transferred to polyvinylidene fluoride (PVDF) membrane (Millipore, Darmstadt, Germany) by electrophoresis. After transfer, the membranes were blocked in PBST buffer containing 5% skimmed milk for 1 h, followed by incubation overnight at 4 °C with 1:2,000 anti-HIF1*α* antibody (ab237544; Abcam, UK) and 1:1,000 anti-*α*-Tubulin antibody (ab7291; Abcam, UK). After multiple washes, the membranes were incubated with horseradish peroxidase conjugated antibody for 1 h at room temperature. Finally, the bands were visualized using an ECL Plus Western Blotting Detection System (GE Healthcare, Little Chalfont, UK).

### Immunofluorescence

MII oocytes from mice were fixed in 4% paraformaldehyde in PBS for 30 min at room temperature, and permeabilized with 0.5% Triton X-100 for 20 min. Following incubation in 1% BSA blocking buffer for 1 h at room temperature, oocytes were incubated with FITC-conjugated anti-Tubulin antibody (F2168; Sigma-Aldrich) at 4 °C overnight. After rinsing with PBS, the oocytes were stained with propidium iodide (PI; 10 µM in PBS). Then the oocytes were mounted on glass slides and examined with a confocal laser scanning microscope (LSM 710; Carl Zeiss, Jena, Germany).

### RNA extraction and Real-time quantitative PCR

Total RNA was extracted from 50 oocytes using the Arcturus PicoPure RNA Isolation Kit (Applied Biosystems, CA, USA). Then RNA from each group was reverse transcribed into cDNA using a QuantiTect Reverse Transcription Kit (Qiagen) according to the manufacturer’s instructions. qRT-PCR was conducted using AceQ qPCR SYBR Green Master Mix (High ROX Premixed) (Vazyme) with Applied Biosystems StepOnePlus Real-Time PCR System (Thermo Fisher Scientific, Massachusetts, USA). Data were normalized against *Gapdh* and quantification of the fold change was determined by the comparative CT method, as previously described (Li et al., 2020). The related primers are listed below: *Hif1α* F: 5′-GCACCGATTCGCCATGGAG-3′, R: 5′-TCTAGACCACCGGCATCCAG-3′; *Hif1α* F: 5′-TCCTTCGGACACATAAGCTCC-3′, R: 5′-GACAGAAAGATCATGTCACCGT-3′; *Hif1α* F: 5′-GAAGTTCACATACTGCGACGA-3′, R: 5′-GTCCAAAGCGTGGATGTATTCAT-3′; *Gapdh*: 5′-AGGTCGGTGTGAACGGATTTG-3′, R: 5′-TGTAGACCATGTAGTTGAGG TCA-3′.

### Statistical analysis

All experiments were repeated at least three times. GraphPad Prism 7 software (GraphPad Software, San Diego, CA, USA) was used to analyze data and draw graphs. Student’s *t* test was used for statistic comparison. Data were reported as mean  ± SD, and *p* < 0.05 were considered statistically significant. n.s., not significant.

## Results

### Generation of mutant mice with oocyte-specific deletion of *Hif1α*

**Figure 1:**
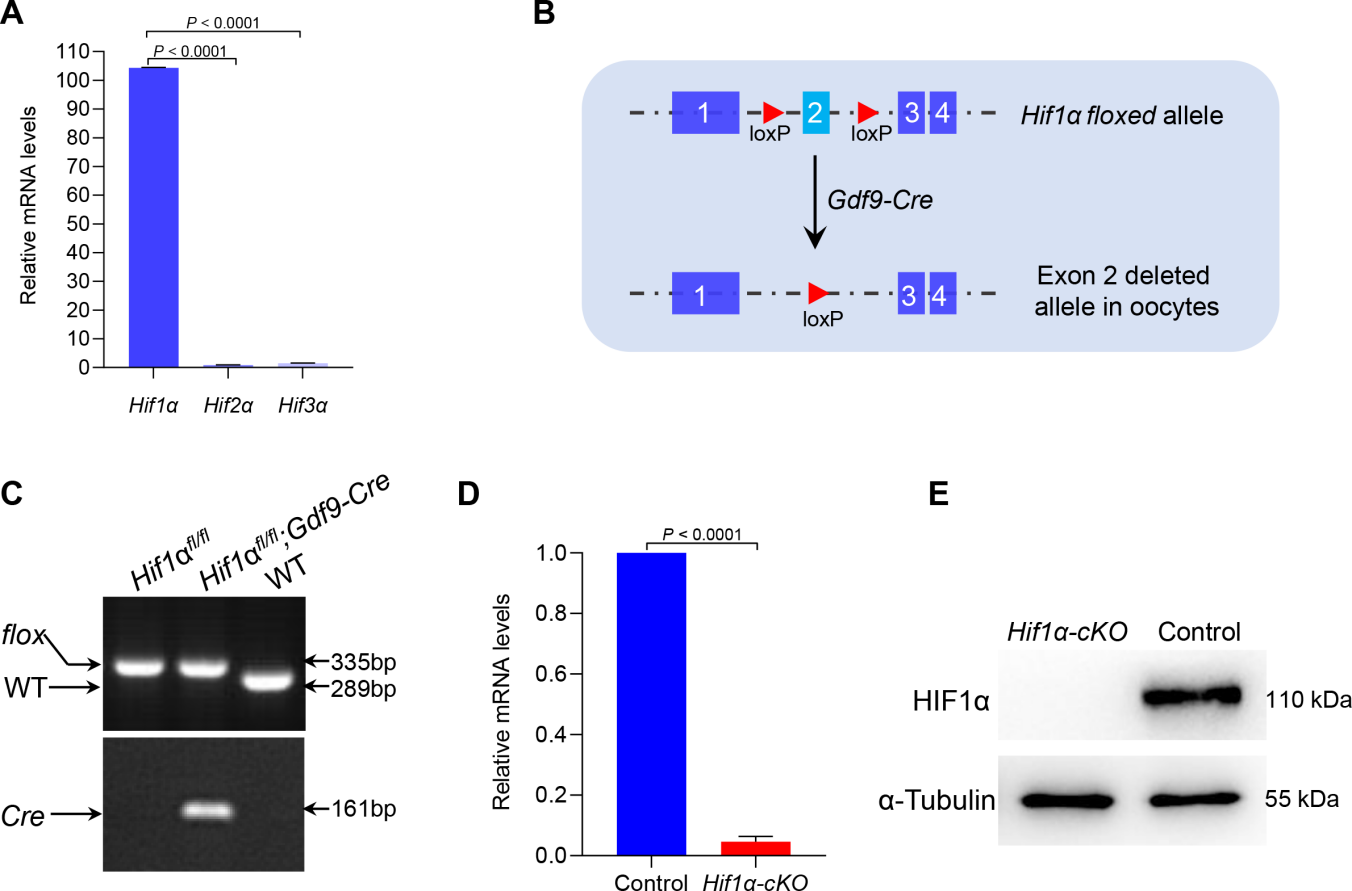
Targeted deletion of the *Hif1α* in mouse oocytes. (A) qRT-PCR analysis of *Hifα* subunits mRNA levels in oocytes from WT mice. The relative mRNA levels of *Hif3α* in WT oocytes were set to 1.0. Data represent the mean ± SD (*n* = 3). (B) Schematic representation of *Hif1α* exon 2 deletion by Gdf9-Cre-mediated recombinase in oocytes. (C) PCR genotyping results of *Hif1α*^*fl*/*fl*^ mice and *Gdf9-Cre* recombinase mice from DNA obtained from tail samples. A single 289 bp band and a single 335 bp band corresponded to the WT and homozygous floxed mice (*Hif1α*^*fl*/*fl*^) respectively (top); a single 161 bp band indicated the *Gdf9-Cre* transgene (bottom). (D) qRT-PCR analysis of *Hif1α* mRNA levels in oocytes from Control and *Hif1α*-*cKO* females. The relative mRNA level of *Hif1α* in Control oocytes was set to 1.0. Data represent the mean ± SD (*n* = 3). (E) Western blot showing the absence of HIF1*α* protein expression in *Hif1α*-*cKO* oocytes. MII oocytes were collected for analysis, and 100 oocytes were used for each sample. Level of *α*-Tubulin was used as an internal control. The experiments were repeated three times. Student’s *t* test (two-tailed) was used for statistical analysis.

So far, three different alpha subunits have been known to exist in higher metazoans, termed HIF1*α*, HIF2*α* and HIF3*α* (Webb, Coleman & Pugh, 2009). By performing quantitative real-time PCR, we showed that *Hif1α* was predominantly expressed in oocytes (Fig. 1A). This observation suggests that HIF1*α* may have an important role in oocyte development. To investigate this, we generated mutant mice in which exon 2 of the *Hif1α* gene was targeted (Fig. 1B). This was achieved by crossing *Hif1α*^*fl*/*fl*^ mice with transgenic mice expressing *Gdf9* promoter-mediated Cre recombinase, which mediates recombination in mouse oocytes at the primordial stage, to knock out *Hif1α* specifically in oocytes (Yu et al., 2013). Hereafter, *Hif1α*^*fl*/*fl*^; *Gdf9-Cre* and *Hif1α*^*fl*/*fl*^ mice are referred to as *Hif1α*-*cKO* and Control mice, respectively. Mice homozygous for this floxed allele and positive for *Gdf9-Cre* transgene were validated by genotyping PCR (Fig. 1C). qRT-PCR confirmed that oocytes from *Hif1α*-*cKO* mice had undetectable *Hif1α* mRNA as compared to those from Control mice (Fig. 1D). Moreover, immunoblotting result showed the absence of HIF1*α* protein in *Hif1α*-*cKO* oocytes (Fig. 1E), suggesting that *Gdf9-* mediated Cre excision of *Hif1α* is sufficient to delete HIF1*α* protein in mouse oocytes.

### HIF1*α* was dispensable for female fertility

**Figure 2:**
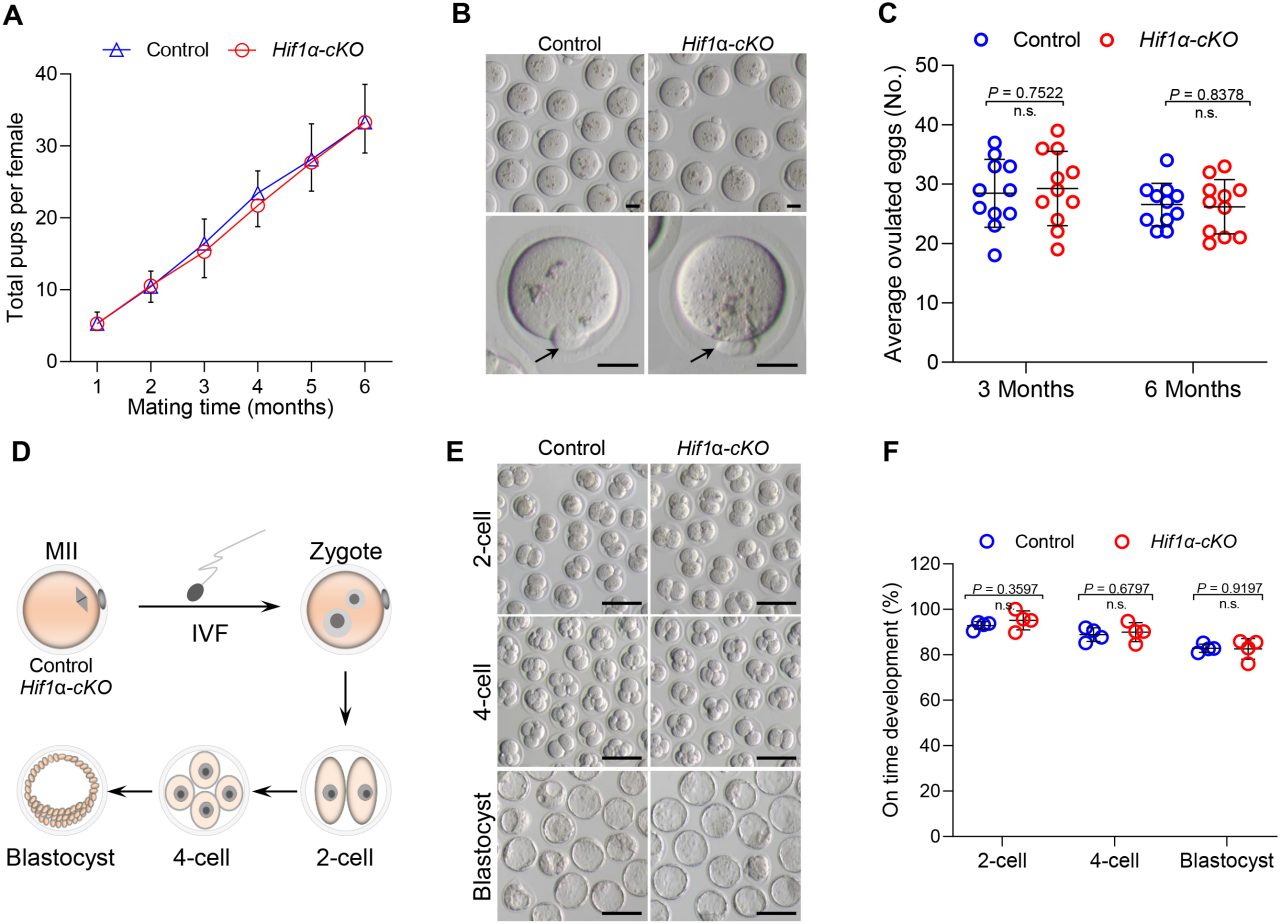
Loss of HIF1 *α* in oocytes had little effects on mouse fertility. (A) Cumulative numbers of pups per female born from Control and *Hif1α*-*cKO* mice for 6 months (*n* = 7). (B) Representative bright-field images of ovulated MII oocytes from Control and *Hif1α*-*cKO* females. Arrows indicate the first polar body. Scale bars = 30 µm. (C) Average number of oocytes obtained after superovulation from Control and *Hif1α*-*cKO* females at indicated age (*n* = 11). (D) Schematic diagram of *in vitro* fertilization and embryo culture. (E) Representative bright-field images of *Hif1α*-*cKO* oocyte-derived E1.5, E2.5 and E4 embryos. Scale bars = 100 µm. Control, *n* = 89; *Hif1α*-*cKO*, *n* = 79. (F) The percentage of *Hif1α*-*cKO* oocyte-derived embryos that successfully progressed to the 2-cell, 4-cell and blastocyst stage at E1.5, E2 and E4 during *in vitro* culture. Data are expressed as mean ± SD. Student’s *t* test (two-tailed) was used for statistical analysis.

To detect the effect of HIF1*α* deletion on female fertility, breeding assay was carried out by mating *Hif1α*-*cKO* or Control mice with males of proven fertility for 6 months. As shown in Fig. 2A, female *Hif1α*-*cKO* mice were fertile and gave birth to pups comparable to that of Control mice. To assess oocyte quality, we superovulated Control and *Hif1α*-*cKO* females and collected oocytes at 16 h post-hCG injection. The oocytes collected from *Hif1α*-*cKO* ovaries were morphologically normal, displaying intact first polar body (Fig. 2B). In addition, there was no difference between the number of ovulated eggs from the Control and *Hif1α*-*cKO* ovaries (Fig. 2C). We then asked whether HIF1*α* deletion in oocytes would adversely affect the developmental competences of subsequent embryos. To do this, we carried out *in vitro* fertilization (IVF) of oocytes derived from Control and *Hif1α*-*cKO* mice, and cultured fertilized embryos *in vitro* to monitor early embryo development (Fig. 2D). Zygotic embryos from *Hif1α*-*cKO* oocytes exhibited similar on-time progression to 2-cell, 4-cell and blastocyst stages relative to Controls (Figs. 2E, 2F). These results show that loss of HIF1*α* in oocytes does not affect the fertility of female mice.

### HIF1*α* deletion exerts little effect on oocyte maturation and spindle organization *in vitro*

**Figure 3:**
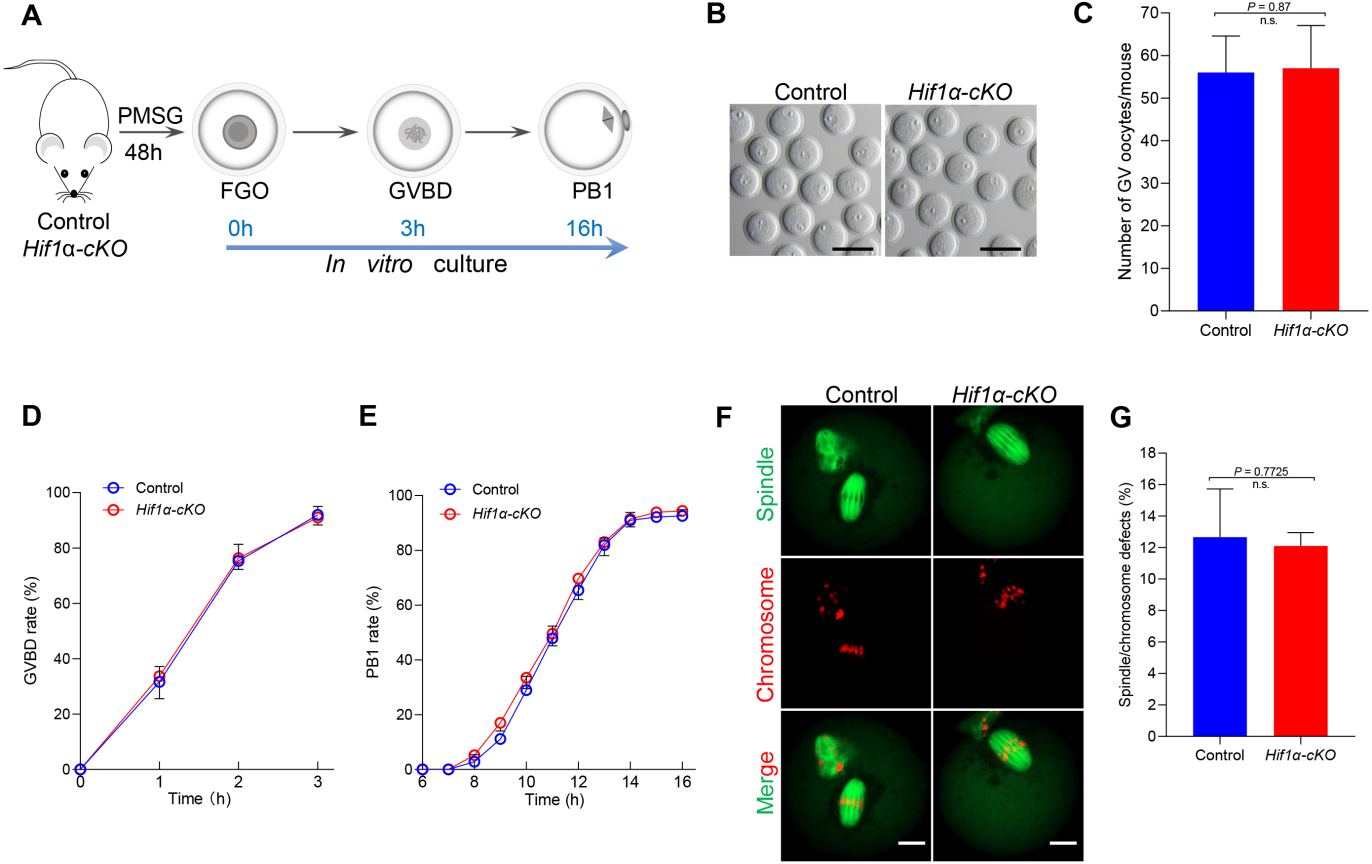
HIF1*α* is not required for oocyte meiotic maturation *in vitro*. (A) Schematic diagram of GV oocyte collection and *in vitro* maturation. (B) Representative bright-field images of GV oocytes collected from Control and *Hif1α*-*cKO* mice. Scale bar = 100 µm. (C) Mean number of GV stage oocytes obtained per mouse after priming with PMSG (*n* = 6 for each genotype). (D, E) GVBD and PB1 rates of oocytes from 8-week-old Control and *Hif1α*-*cKO* mice. Control, *n* = 67; *Hif1α*-*cKO*, *n* = 84. (F) Oocytes from Control and *Hif1α*-*cKO* mice were immunolabeled with *α*-Tubulin to label spindle (green) and co-stained propidium iodide to visualize chromosomes (red). Scale bar, 20 µm. (G) Quantification of Control (*n* = 78) and *Hif1α*-*cKO* oocytes (*n* = 69) with spindle defects or chromosome misalignment. Data are expressed as mean ± SD. Student’s *t* test (two-tailed) was used for statistical analysis. n.s., not significant.

Given that HIF1*α* is not essential for oocyte maturation in vivo, we next studied how *Hif1α*-*cKO* oocytes matured *in vitro*. Fully-grown oocytes harvested from hormonally stimulated Control and *Hif1α*-*cKO* mice were cultured in maturation media to assess meiotic resumption within 3 h, and PB1 extrusion within 16 h (Fig. 3A). Comparable numbers of fully-grown germinal vesicle-stage (GV) oocytes were obtained from Control and *Hif1 α*-*cKO* mice ovaries (Figs. 3B, 3C), suggesting that the ovarian reserve is not compromised in the absence of HIF1*α*. Then, the collected GV oocytes were released for maturation *in vitro*. Meiotic resumption, as indicated by GV breakdown (GVBD), occurred in 80% of *Hif1α*-*cKO* oocytes during the first 2 hours’ culture, similar to the rate observed in Control oocytes (Fig. 3D). Next, we analyzed the exit from MI, marked by first polar body extrusion (PBE). We found that PBE began around 8–10 h for both Control and *Hif1α*-*cKO* oocytes and that both attained maximal PB1 rates of around 95% by 14 h (Fig. 3E). In addition, we examined spindle organization and chromosome alignment in Control and *Hif1α*-*cKO* oocytes that had extruded PB1. Immunofluorescence results showed that barrel-shape spindle and well-aligned chromosomes were readily observed in *Hif1α*-*cKO* oocytes, and that errors in meiotic apparatus were not markedly increased (Figs. 3F, 3G). Thus, loss of HIF1*α* does not compromise oocyte maturation and spindle organization *in vitro*.

### Loss of HIF1*α* did not disrupt the expression pattern of other HIF isoforms in oocytes

**Figure 4:**
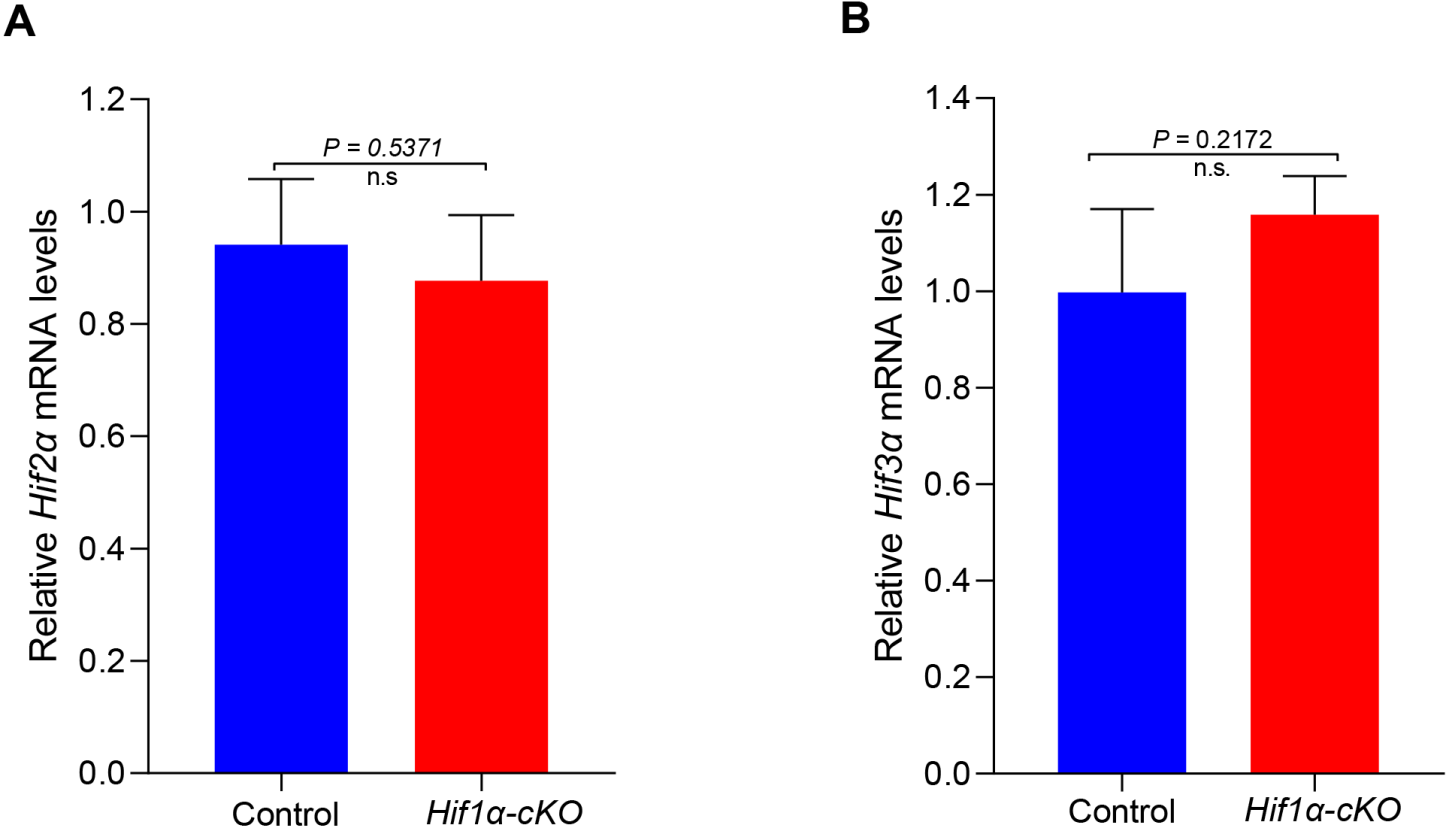
Expression analysis of other *Hifα* subunits in Control and *Hif1α*-*cKO* oocytes. Relative mRNA levels of *Hif2α* and *Hif3α* are determined by real-time RT-PCR (*n* = 3). Data are expressed as mean ± SD. Student’s *t* test (two-tailed) was used for statistical analysis. n.s., not significant.

HIF*α* subunits exist as a series of isoforms encoded by distinct genetic loci. Among three HIF*α* isoforms, HIF1*α* and HIF2*α* appear closely related and can interact with hypoxia response elements (HREs) to activate transcription (Wiesener et al., 1998). We speculated that other subunits might compensate for the role of HIF1*α* in oocytes. However, the mRNA expression levels of *Hif2α* and *Hif3α* were not significantly changed in HIF1*α*-deleted oocytes (Fig. 4). This finding indicates that the null of HIF1*α* did not induce the compensatory expression of other isoforms, and demonstrates that HIF pathway is not required for oocyte development.

## Discussion

The adaptation of cells to the anaerobic environment is achieved by the transcriptional induction of genes that are involved in glycolysis, haematopoiesis, angiogenesis, invasion and regulation of vascular tone (Kaelin Jr & Ratcliffe, 2008). An evolutionarily conserved pathway mediated by oxygen-dependent posttranslational hydroxylation of a transcription factor called hypoxia-inducible factor (HIF) plays a pivotal role in this process (Giaccia, Siim & Johnson, 2003). However, because its deletion in mice causes embryonic lethality (Carmeliet et al., 1998), the *in vivo* roles of the HIF pathway in reproduction remain unclear. Our findings showed that *Hif1α* isoform was highly expressed in oocyte (Fig. 1A). We then constructed mice with oocyte-specific knockout of *Hif1α* at growing oocyte to investigate the potential role of *Hif1α* in oogenesis. Surprisingly, we found that HIF1*α* is dispensable for ovulation and female fertility in mice. Moreover, oocyte can mature *in vitro*, and form well-organized spindle in the absence of HIF1*α* (Fig. 3). In addition, it has been reported that other HIF*α* subunits appear closely related and are able to interact with hypoxia response elements (HREs) to induce transcriptional activity (Webb, Coleman & Pugh, 2009). However, we did not observe pronounced differences in *Hif2α* and *Hif3α* expression between Control and *Hif1α*-*cKO* oocytes (Fig. 4). Thus, our study shows that HIF pathway in oocyte is not required for female fertility.

It has been long thought that oocytes within antral follicles are limited access to oxygen. However, data accurately reflecting the O_2_ concentration adjacent to an oocyte are lacing (Thompson et al., 2015). A study has shown that oocytes within antral follicles exist in a relatively low oxygen tension, about 11–51 mm Hg (Redding, Bronlund & Hart, 2008). However, considering the potential for low O_2_ concentration within antral follicles, there is a paucity of evidence that HIF is involved in mammalian oogenesis and folliculogenesis. By developing a HIF reporter mouse, researchers found no HIF reporter activity in mural granulosa, cumulus layers, or oocytes from preovulatory follicles (Tam et al., 2010). This suggests HIF activity is dispensable for the development of growing antral follicle. Here, by using oocyte-specific HIF1*α* deletion mice, we further demonstrated this notion. There exists a paradox for the developing follicle. One possible explanation is that a low but not hypoxic environment exists in the follicle, most likely for protecting the oocyte from oxidative damage while offering enough O_2_ to meet its oxidative phosphorylation demands. Meanwhile, following the LH (luteinizing hormone) surge, HIF activity is dramatically increased and plays a key role in luteinization (Kim, Bagchi & Bagchi, 2009). In addition, echinomycin, an HIF1*α* inhibitor significantly decreased bovine oocyte maturation and subsequent blastocyst formation in vitro through modulating cumulus cell function (Turhan et al., 2021). Thus, more studies are required to determine the significance of HIF pathway in granulosa cells.

## Conclusions

In conclusion, by generating mutant mice with oocyte-specific deletion of HIF1*α*, we provided genetic evidence that the HIF pathway in oocyte is not required for ovulation and female fertility. Our results further support the notion that oocyte resides in follicle with a low O_2_, but not a hypoxic environment.

## Supplemental Information

10.7717/peerj.13370/supp-1 Supplemental Information 1 Raw data of Hif*α* subunits expression in oocytes qRT-PCR analysis of *Hifα* subunits mRNA levels in oocytes from WT mice. The relative mRNA levels of *Hif3α* in WT oocytes were set to 1.0. Data represent the mean ± SEM (*n* = 3)

10.7717/peerj.13370/supp-2 Supplemental Information 2 Raw data for PCR genotyping PCR genotyping results of *Hif1α*^*fl*/*fl*^ mice and *Gdf9-Cre* recombinase mice from DNA obtained from tail samples. A single 289 bp band and a single 335 bp band corresponded to the WT and homozygous floxed mice (*Hif1α*^*fl*/*fl*^) respectively (Top); a single 161 bp band indicated the *Gdf9-Cre* transgene

10.7717/peerj.13370/supp-3 Supplemental Information 3 Raw data of *Hif1α* expression in cKO oocytes. qRT-PCR analysis of *Hif1α* mRNA levels in oocytes from Control and *Hif1α*-*cKO* females. The relative mRNA level of *Hif1α* in Control oocytes was set to 1.0.

10.7717/peerj.13370/supp-4 Supplemental Information 4 Raw data for WB Western blot showing the absence of HIF1*α* protein expression in *Hif1α*-*cKO* oocytes.

10.7717/peerj.13370/supp-5 Supplemental Information 5 Raw data for fertility test Cumulative numbers of pups per female born from Control and *Hif1α*-*cKO* mice for 6 months.

10.7717/peerj.13370/supp-6 Supplemental Information 6 Raw data for superovulation Average number of oocytes obtained after superovulation from Control and *Hif1α*-*cKO* females at indicated age

10.7717/peerj.13370/supp-7 Supplemental Information 7 Raw data for early embryo on time development The percentage of *Hif1α*-*cKO* oocyte-derived embryos that successfully progressed to the 2-cell, 4-cell and blastocyst stage at E1.5, E2 and E4 during *in vitro* culture.

10.7717/peerj.13370/supp-8 Supplemental Information 8 Raw data for the number of GV oocyte Mean number of GV stage oocytes obtained per mouse after priming with PMSG (*n* = 6 for each genotype).

10.7717/peerj.13370/supp-9 Supplemental Information 9 Raw data for GVBD rate GVBD rates of oocytes from 8-week-old Control and *Hif1 α*-*cKO* mice.

10.7717/peerj.13370/supp-10 Supplemental Information 10 Raw data for PB1 rates PB1 rates of oocytes from 8-week-old Control and *Hif1 α*-*cKO* mice.

10.7717/peerj.13370/supp-11 Supplemental Information 11 Raw data for meiotic defects Quantification of Control (*n* = 78) and *Hif1α*-*cKO* oocytes (*n* = 69) with spindle defects or chromosome misalignment. Data are expressed as mean ± SD.

10.7717/peerj.13370/supp-12 Supplemental Information 12 Raw data for *Hifα subunits expression* Relative mRNA levels of *Hif*2*α* and *Hif3α* are determined by real-time RT-PCR. Data are expressed as mean ± SD.

10.7717/peerj.13370/supp-13 Supplemental Information 13 Author checklist

## Additional Information and Declarations

### Data Availability

The following information was supplied regarding data availability: The raw measurements are available in the Supplementary File.
