# Supplementary figures and images for: HIF1α is dispensable for oocyte development and female fertility in mice
Source: PeerJ. 2022 May 3;10:e13370. doi: 10.7717/peerj.13370 (PMC9074875; doi:10.7717/peerj.13370)

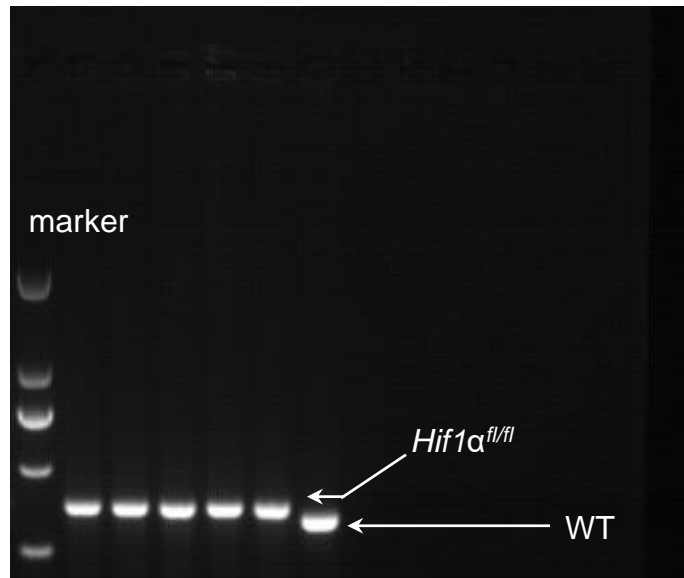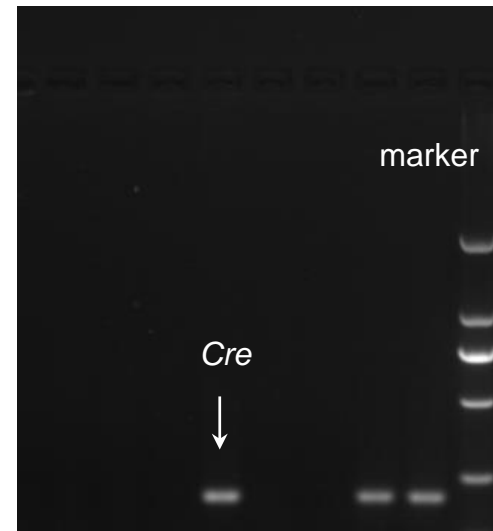

Supplement: Supplemental Information 2 — PCR genotyping results of Hif1αfl/fl mice and Gdf9-Cre recombinase mice from DNA obtained from tail samples. A single 289 bp band and a single 335 bp band corresponded to the WT and homozygous floxed mice (Hif1αfl/fl) respectively (Top); a single 161 bp band indicated the Gdf9-Cre transgene [file peerj-10-13370-s002.pdf]

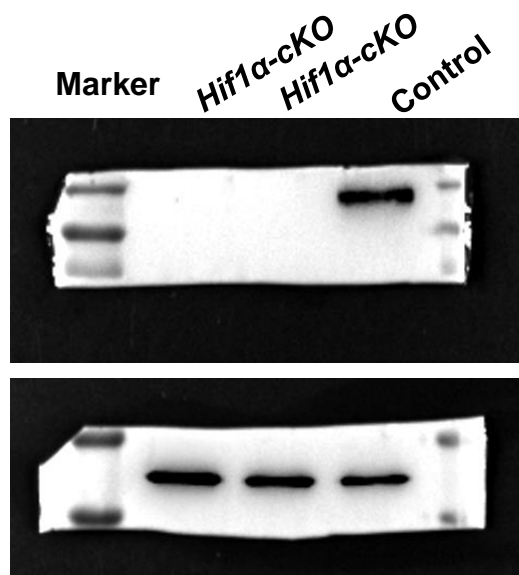

HIF1 $\alpha$

$\alpha$ -Tubulin

Supplement: Supplemental Information 4 — Western blot showing the absence of HIF1α protein expression in Hif1α-cKO oocytes. [file peerj-10-13370-s004.pdf]
